# An Improved CCF Detector to Handle the Problem of Class Imbalance with Outlier Normalization Using IQR Method

**DOI:** 10.3390/s23094406

**Published:** 2023-04-30

**Authors:** Amerah Alabrah

**Affiliations:** Department of Information Systems, College of Computer and Information Sciences, King Saud University, Riyadh 11451, Saudi Arabia; aalobrah@ksu.edu.sa

**Keywords:** credit card fraud, feature selection, outliers, oversampling, SMOTEN

## Abstract

E-commerce has increased online credit card usage nowadays. Similarly, credit card transactions have increased for physical sales and purchases. This has increased the risk of credit card fraud (CCF) and made payment networks more vulnerable. Therefore, there is a need to develop a precise CCF detector to control such online fraud. Previously, many studies have been presented on CCF detection and gave good results and performance. However, these solutions still lack performance, and most of them have ignored the outlier problem before applying feature selection and oversampling techniques to give solutions for classification. The class imbalance problem is most prominent in available datasets of credit card transactions. Therefore, the proposed study applies preprocessing to clean the feature set at first. Then, outliers are detected and normalized using the IQR method. This outlier normalizes data fed to the Shapiro method for feature ranking and the 20 most prominent features are selected. This selected feature set is then fed to the SMOTEN oversampling method, which increases the minority class instances and equalizes the positive and negative instances. Next, this cleaned feature set is then fed to five ML classifiers, and four different splits of holdout validation are applied. There are two experiments conducted in which, firstly, the original data are fed to five ML classifiers and the holdout validation technique is used, in which the AUC reaches a maximum of 0.971. In Experiment 2, outliers are normalized, features are selected using the Shapiro method, and oversampling is performed using the SMOTEN method. This normalized and processed feature set is fed to five ML classifiers via holdout validation methods. The experimental results show a 1.00 AUC compared with state-of-the-art studies, which proves that the proposed study achieves better results using this specific framework.

## 1. Introduction

Online shopping has increased online payments via websites. In this way, credit card information has become more vulnerable to attackers [1]. Credit-card-based fraud has increased exponentially in recent years [2]; to control such activities, the transaction process should be fraudless. Therefore, there is a need to install a fraudulent behavior detector [3] during credit card transaction steps.

Fraud is an individual act of falsehood in any cyberspace [4]. It basically consists of two types, online and offline. The offline type is controlled by law enforcement agencies such as the police, etc. However, online fraud is a broad term as it includes the complete world. Online fraud takes place in different ways, such as stealing credit card information, the misuse or sharing of confidential data [5], fraudulent accounts, scam emails, and phishing [6], etc. There are some traditional methods used to steal information from online purchasers, such as obtaining their card information, names, CCV codes, secret pins, etc. Similarly, merchant-side fraudulent schemes include offers of discounts on different items, which convinces the user to share their credit card information. Further, some greedy-algorithm-based credit card information creation methods are also applied on some E-commerce sites to purchase items, and in some cases, it is successful [7]. Electromagnetic chips are also installed on machines to read card information [8].

Due to these online fraud attacks, millions of dollars and other organizational losses have occurred in recent years [9]. Therefore, a fraudulent activity detector is needed that is developed to detect the unusual, suspicious behavior of scammers. In credit card fraud (CCF) detection, machine learning techniques and tools are used. Many previous studies have been performed on CCF but they still present many challenges, such as the overfitting of machine learning methods, the imbalance of class data [10], and the continuously changing behavior and patterns of fraudsters [11,12]. Similarly, the outliers found in big datasets are also a problem as they cause the inconsistent behavior of transaction records and could be detected as suspicious activity, whereas it reflect the normal activity of a network transaction. However, outliers found in a consistent flow of transaction records could be normalized or removed before applying any class balancing on the minority class or using classification techniques. It was stated in a report that the consistent behavior of large datasets does not change over time, whereas the outliers found in them need to be detected and solved appropriately [13]. Furthermore, the given datasets for the development of CCF have a huge data imbalance problem. The class imbalance arises when the improper distribution of instances occurs against each category. In this way, highly skewed data are most probably biased towards majorly normal class instances [3]. This improper distribution of datasets compromises the performance of CCF, as, if the ML model is trained at 95% on a single class pattern, then it will most probably detect a fraudulent transaction pattern as a normal transaction.

Therefore, minority class observations need to be considered in CCF model training to be as important as normal class observations. The class imbalance problem was solved previously in many ways, such as by class oversampling [14], algorithm-based synthetic data generation [15], and resampling [16]. Data balancing obtains appropriate input data for ML model training, which leads to more confidence and performance efficiency in applied approaches. The CCF training data already have thousands of instances, while, in a class imbalance situation, the dataset becomes larger when any technique of class balancing is applied. In this way, if machine learning data are big, this can cause overfitting, even after the class balancing of all categories. Therefore, the removal of useless features is also an important factor to obtain a more efficient CCF detection system [17]. CCF detection systems have also been proposed previously with different feature selection techniques, such as adaptive feature selection [18], information-gain-based high-weight feature selection [19], and other techniques. However, the feature selection in these methods is still performed manually to select features based upon their higher performance, such as selecting the highest-ranked 10 or 15 features, etc.

Therefore, feature selection needs to be optimized to select the highly important features automatically from the given dataset. This will led to the development of a fully automated CCF detection system containing a class-balanced dataset with a highly contributed feature-selection-based solution. However, the classification of normal and fraudulent transactions is performed on both ML and DL models. These models mostly have two types of applied approaches, supervised and unsupervised machine learning methods, which are used to identify the normal and fraudulent behavior of credit card transactions [20]. The supervised methods use already available data containing labels of fraudulent data or normal transaction data. The unsupervised method uses unlabeled data and only obtains clusters on the unusual behavior of transaction records [12]. The supervised methods are most useful for activities that take place based upon the previous records of fraud, whereas unsupervised methods are useful to detect new types of malicious attacks. The present work contributes in the following ways:

- The noisy outliers are normalized from the feature set using the IQR method and normalized all features;
- The Shapiro method of feature selection is used on an outlier-free feature set to make the feature set more appropriate, smaller in size, and noise-free;
- The SMOTEN method is applied to address the class imbalance problem on the preprocessed cleaned feature set;
- The holdout validation method is used to validate the performance of the proposed feature set processing-based CCF detector.

The rest of the article is divided into four parts. Section 2 offers a recent study review regarding the CCF detection system. Section 3 describes the applied method to solve the efficient CCF detection system development problem, and Section 4 provides the results and discussion to validate the results of the applied approach. Lastly, a comparison with state-of-the-art studies shows the robustness of the proposed method. The conclusion, including limitations and future directions, is given at the end.

## 2. Related Work

Artificial-intelligence-based solutions are continuously solving real-time problems such as in edge computing [21], time series forecasting [22], and object tracking using deep learning methods [23], and similarly offer different types of solutions in credit card fraud detection, such as meta-heuristic approaches [24]. Hence, many studies have been performed previously on CCF development to solve the class minority and feature selection problems. Most of them use random oversampling and undersampling techniques. The authors of [25] used undersampling, oversampling, SMOTE, and random oversampling techniques, whereas SMOTE was considered to be the best, as it generated more optimal synthetic data to balance the dataset. The dataset provided by Kaggle is used in this study, which contains only 492 instances of fraudulent and 24,315 instances of normal transactions. Five different ML classifiers are applied for classification. Logistic regression achieves the highest accuracy, Area Under Curve (AUC), and recall scores of 97.04%, whereas precision is 99.99%. The class balancing problem is solved using the Synthetic Minority Class Oversampling Technique (SMOTE), a method of oversampling the instances for the minority class [26]. We can see that the class minority problem is solved but outlier removal is not performed at first, due to which the noisy data are not excluded and minority-class-based new instances have been created. A summary of recently applied CCF detection methods has been shown in Table 1.

The Genetic Algorithm (GA) method is applied for feature selection on oversampled data. The traditional ML methods of classification, such as Naïve Bayesian, decision tree, logistic regression, and random forest, are applied. The same dataset as used by the previous study is used in this study, whereas only seven features have been selected from 42 features after applying the GA method. However, after applying the synthetic method of dataset oversampling, the accuracy and other measures are calculated and found to be improved, with the highest results reaching up to 99.96% for the LR method, with 99.12% recall, 80.68% precision, and 88.98% F1-score. However, using the RF method as a classifier, the accuracy is slightly decreased by 99.95%, whereas other measures are improved, such as recall as 99.82%, as well as 99.92% precision and an F1-score up to 99.82%. It illustrates the strength of the feature selection method with the use of the oversampling technique. However, feature selection is still a manual scheme in this study, which needs to be automated. Moreover, outlier removal is not applied.

Another study stated that the class imbalance problem is mostly solved but the class overlap problem remains unsolved [30]. The anomaly-detection-based method is applied to minority and majority samples, which excludes the outliers from the original dataset. A non-linear classifier is applied to better distinguish the overlapping solved dataset. However, a dynamic weighted entropy-based metric is proposed to measure the excluded overlapping outliers’ performance. The Kaggle dataset and other real electronic datasets of credit card transactions are used and various experiments are performed. This study applies outlier removal but it removes them, whereas data are collected on a real-time basis. By excluding real data, we will lose data, whereas some form of normalization technique could be applied to utilize all transactions’ records.

Z. Zhang et.al [27] used a Convolutional Neural Network (CNN) and Auto-Encoder methods to classify fraudulent and non-fraudulent activities. A new dataset of fraudulent activities and the Kaggle dataset are used for fraudulent activity identification. Random undersampling and oversampling using SMOTE method are applied to the datasets. These minority and majority class-balanced datasets are fed to the CNN and Auto-Encoder networks for training and testing. The classifiers tested on the Kaggle dataset showed 93% accuracy but did not perform well for another private dataset. This makes this study less robust as a generic approach cannot be applied to all datasets. Furthermore, feature selection and outlier removal were also not applied. A different type of approach is applied in [28], in which text-to-image transformation is applied on the Kaggle dataset; this image dataset is then fed to a CNN classifier and deep features are extracted. The CNN and ML classifiers are adopted for the classification of the Kaggle dataset. To solve class imbalance, the class weights are assigned while feeding to DL and ML classifiers. The maximum achieved accuracy is 99.87% using KNN-Coarse. The outlier removal and feature selection are not applied, and the class imbalance problem is also not solved before applying classification.

S. Tamtama [29] proposed a framework that includes data transformation using PCA for categorical to numeric data transformation, with random over- and undersampling with SMOTE, and classification using the random forest method applied. The Data Card Fraud of Europe dataset, which is also the Kaggle dataset, is used, and classification accuracy of 99.99% is achieved with a 70–30 split of data. The applied study uses only a random forest classifier, accuracy, and a confusion matrix for results evaluation.

Further, multiple splits of training and testing are not applied for further checking of the applied approach’s robustness. Although the accuracy is very high, the F1-score and AUC measures relate to minority class-based performance, but they are not provided or not improved, as the class imbalance problem is not solved before applying the classification.

The most recent studies have been discussed in detail with the most used datasets, where the applied approaches use different methods of oversampling and undersampling to solve the class imbalance problem. The most adopted methods include SMOTE and random sampling of minority and majority class sample balancing. Further, it is noticed that Kaggle and other real-time-collected private datasets are used. However, all of them have class imbalance problems, where the metrics are not used appropriately, while the class-based results need to be shown. Many of the studies use accuracy, precision, recall, etc.

These measures should be extended with the use of the F1-score and AUC score, which specifically reflect the performance of the applied methods by targeting the class-based results. Therefore, there is a need to apply an appropriate method for class balancing, the class overlapping problem, and the classification problem with the evaluation of relevant metrics. Further, validation methods should be bias-free and should include multiple splits and folds to test and train the oversampled and undersampled datasets.

## 3. Materials and Methods

**Figure 1:**
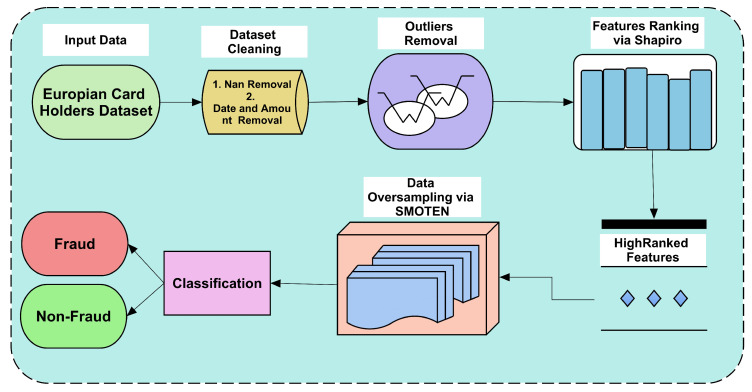
Applied framework to improve performance of CCF detection systems.

The applied framework uses different techniques to develop an efficient method to form a CCF detection system. We first took a highly imbalanced dataset with minority class instances being very low as compared to majority class instances. All steps are shown in Figure 1, which reflects the scheme of the applied framework.

The dataset is cleaned by removing null rows and instances; secondly, the outliers from the dataset are checked and normalized via the IQR method. The IQR method is abundantly applied in many other fields to improve the quality of applied methods as it normalizes data. Improved versions of the IQR method are also proposed [31], which have slight variations in calculations. Thirdly, the most prominent features are selected by ranking them using the Shapiro method. The selected feature set is used to create an oversampled dataset. The oversampled feature set is used to classify fraudulent and non-fraudulent instances. There are two experiments performed in the classification. In Experiment 1, the cleaned dataset is used to classify using ML classifiers, and 4 different splits of the dataset are used. In Experiment 2, the outliers are normalized, the features selected, and the oversampled dataset is used to classify the dataset.

### 3.1. Dataset Cleaning

The dataset is cleaned using the removal of null values or instances. The instances that contain any unavailable values are considered to be removable. Furthermore, data cleaning is considered to remove the less contributing features by checking them with the Shapiro method of feature ranking. The features that do not correlate with the output variable are removed. The mathematical representation is shown in Equation (1).

(1) $$f\left(x\right)\leftarrow d_{i}=0,x_{i}\leq 0$$

The date and amount variables from the dataset are removed, as the date variable or feature does not play an important role in the CCF detector system. Similarly, the amount depends upon the account balance. Whenever an attack is performed on an account, the amount will be deducted. Therefore, these two column features are removed before applying the next preprocessing steps.

### 3.2. Outlier Normalization Using IQR Method

**Figure 2:**
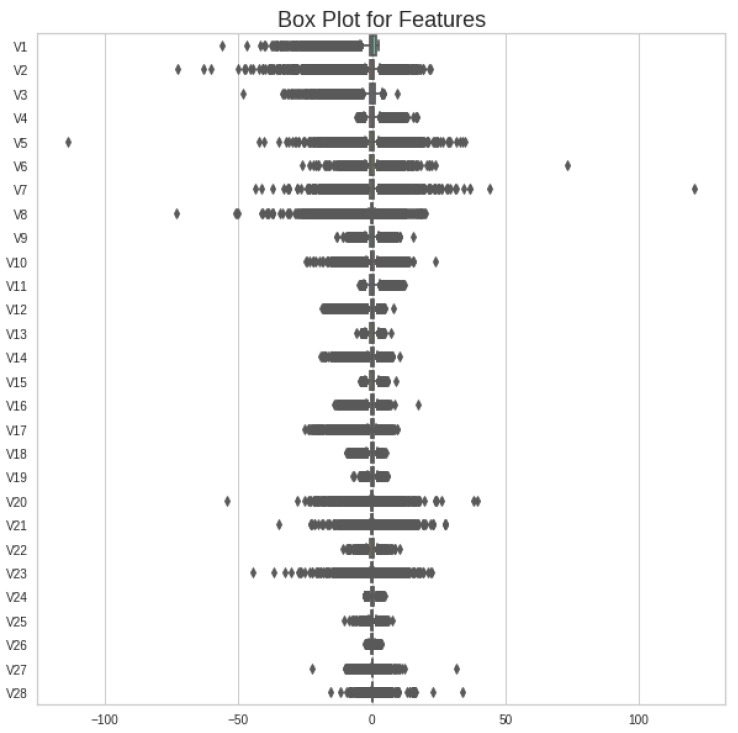
Boxplot of all features of original Kaggle CCF dataset showing quartile range of each feature.

In any given dataset, if we compare the outlier’s range against each feature, then we find certain values that are extremely out of range as compared to most of the instances found in the feature column. These values mislead any machine learning classifier during training and may change the range of incoming testing values after deployment. Therefore, the removal of these values is necessary before moving to the next steps. To check the range of values against each feature, a boxplot is used to visualize the quartile ranges of all features, as shown in Figure 2.

Most of the features have a certain range of input values in the whole dataset, whereas a few of them show extreme values or outliers, which need to be replaced with an appropriate method. However, outlier removal is adopted using the Inter-Quartile Range (IQR) method. The IQR method’s mathematical range is represented in Equation (2).

(2) $$IQR\;=\;q_{3}-q_{1}$$

**Figure 3:**
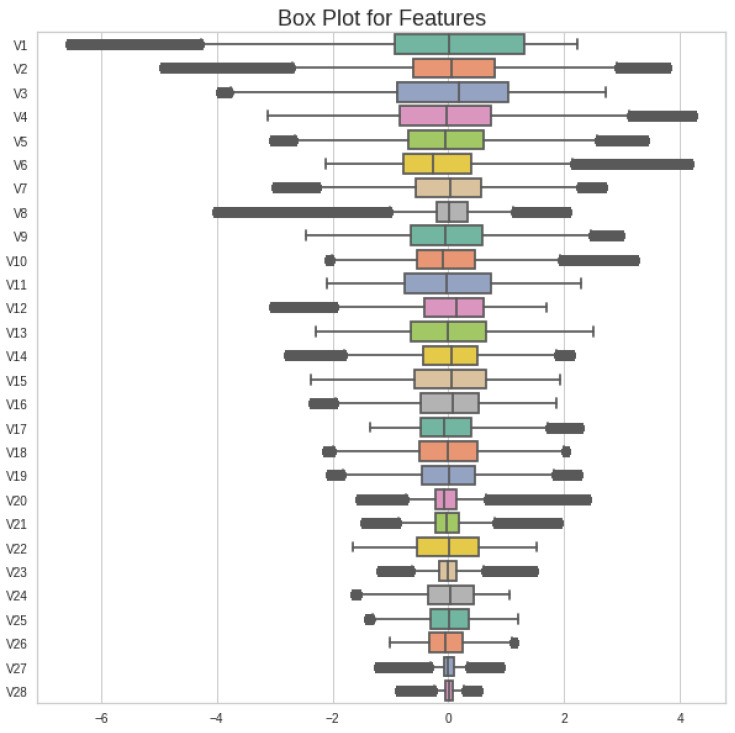
Boxplot of capped values of all features of original dataset using IQR method.

The IQR, which is the resulting value, will be adopted to cover the feature outlier values; it is the 75th percentile of all data values. It is calculated by arranging the data values in ascending order and then dividing them into 4 equal parts or quartiles. Then, the 1st and 3rd quartiles are found and, with their subtraction, we find an inner range of values called the IQR value. These inner values are adopted for the whole dataset and we cap all values. The dataset after applying the IQR method is as shown in Figure 3.

The values of all features after capping them using the IQR method are expanded. These values make all values of the dataset normalized. There are a few features that contain outliers, but we apply the IQR method to all features to make all datasets normalized. This makes the dataset bias-free and it is distributed in equal ranges.

### 3.3. Feature Ranking via Shapiro Method

**Figure 4:**
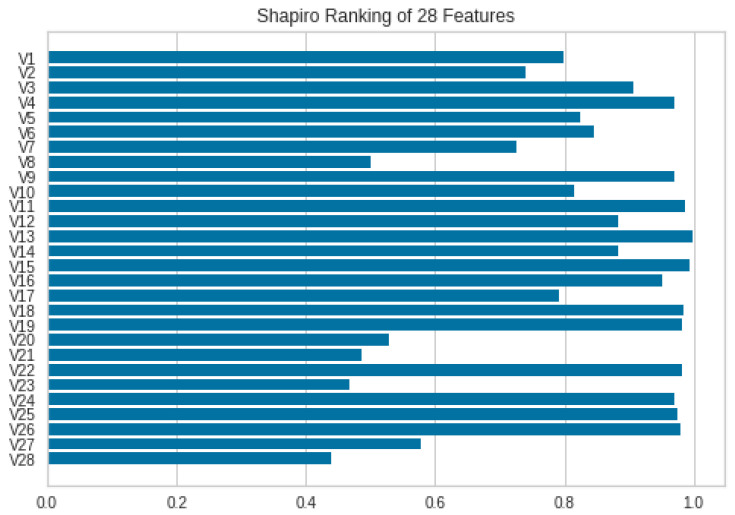
Shapiro ranking of all normalized features.

The normalized dataset is finalized to proceed with classification. However, the removal of redundant or less meaningful features is adopted for feature selection. There are many feature selection methods used in previous studies; in the proposed work, many of these methods were used to classify the data but the Shapiro method was found to be the best among them. Therefore, it is finalized for further classification. The feature ranking is shown in Figure 4.

The different features are ranked as more than 60%, and some of them are even ranked 100%. Therefore, the most important ones, which include scores of more than 50%, are adopted and used for the feature subset of important features. The Shapiro method’s functionality is represented in Equation (3).

(3) $$f\left(x\right)\leftarrow \left(\frac{\Sigma_{t=1}^{n}c_{t}y_{t}}{\Sigma_{t=2}^{n}x_{t}{}_{t}}\right)$$

The function f(x) is used to calculate a statistical value from a fraction, which uses *t* = 1 to n for all values of the table by multiplying the ordered values $\left(y_{t}\right)$ with coefficient values $\left(c_{t}\right)$, and then we divide them with other values of the table shown as $x_{t}$ and ${}_{t}$. The computed value will be considered as the ranking of features.

### 3.4. Data Oversampling via SMOTEN

SMOTE is a synthetic means to oversample a given dataset. It increases the minority class dataset into many new optimal instances. However, many variations of the SMOTE method have been proposed previously, which increase the quality of newly created data. Specifically, SMOTEN is a hybrid variation of the SMOTE method. It not only performs oversampling but also deletes the nearer instances or data from the majority class. It works in such a way that the nearest neighbors of the majority class are estimated and deleted, which excludes the most extensive data before performing oversampling of the minority class.

Therefore, as the dataset has thousands of data for the majority class, there will be adding further noise in their instances too, which is removed at first, and then the minority class oversampling is performed.

### 3.5. Classification Using ML

The classification methods are used from the ML domain and results are calculated to estimate the performance of the applied methods. There are 5 classifiers, named linear regression (LR), Gaussian Naïve Bayesian (GNB), K-nearest neighbor (KNN), decision tree (DT), and random forest (RF), whereas 4 different ratios of data for training and testing are used. There are two experiments presented in the next section. In Experiment 1, data classification on the original cleaned dataset is performed using 5 different dataset training and testing splits. In Experiment 2, data classification results for outlier removal, feature selection, and oversampled datasets using 4 different dataset splits are presented.

## 4. Results and Discussion

The applied framework uses certain methods to obtain an efficient and promising CCF. There are two main experiments that have been performed, which have been conducted to give a performance comparison. The comparison is based on the results before and after applying the techniques to solve the problems. In Experiment 1, five different ML classifiers are used and applied to the cleaned original dataset, whereas four different data splits are used. In Experiment 2, the same classifiers and data splits are used for outlier normalization, feature selection, and the oversampled feature set. The most often used performance evaluation metrics are used and compared with state-of-the-art studies.

### 4.1. Dataset Description

The dataset used in this study is highly imbalanced, as discussed in the Introduction. The normal and fraudulent class distributions are very different. The number of normal and fraudulent instances is shown in Table 2. Furthermore, the dataset distribution after applying SMOTEN oversampling is also included in Table 2.

It is seen that there is a highly class-imbalanced ratio for the original dataset, which has only 492 fraudulent instances. However, for outlier normalization and selected feature data, oversampling is performed on minority class instances and leads to an equivalent number of samples.

### 4.2. Experiment 1: Classification Results before Applying Outlier Normalization, Feature Selection, and Oversampling

Five different ML classifiers are used for the classification of cleaned features’ data. The holdout scheme of validation is used with four different splits of data. The 80–20, 70–30, 60–40, and 50–50 splits of data are used, in which 80, 70, 60, and 50% are training data, whereas 20, 30, 40, and 50% are testing data, taken equally from both categories of the original dataset. Classification results of all classifiers are shown in Table 3.

In the first split, 80–20, 80% of the data were used for training and 20% for testing. However, the performance of all classifiers showed slight variations. The highest performer could be taken based on the Area Under Curve (AUC), which varies strongly among all classifiers. We can see that GNB is the lowest one among all classifiers’ performance in terms of accuracy and kappa coefficient, whereas, in terms of AUC, the DT is the lowest one. If we look at the performance of all five classifiers and their different metric scores, we can see that, except GNB, all classifiers show results of more than 99%. However, for these decimal places based on similar scores, we cannot confirm the better performance of any classifier. In addition, the AUC value showed different behavior as the LR’s AUC is 0.968, and GNB’s AUC is 0.964, which is very much similar, but, in terms of accuracy, it changes from 99% to 97%. The best AUC score with an 80–20 split of data is 0.968 for the LR method of classification.

**Figure 5:**
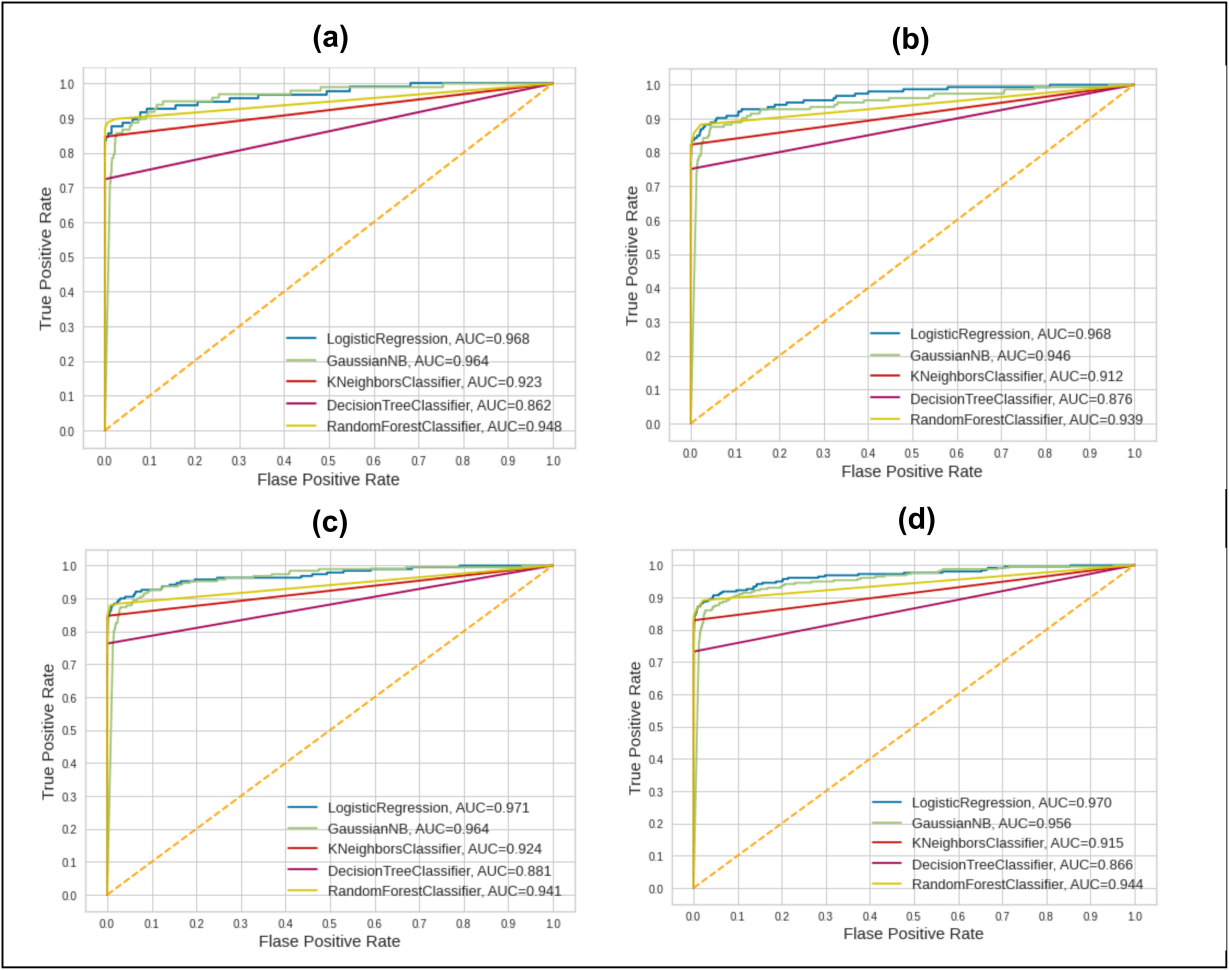
AUC Scores of 5 ML classifiers before applying proposed framework techniques on 4 splits of data. (**a**) 80–20; (**b**) 70–30; (**c**) 60–40; (**d**) 50–50.

In the second split of data, 70% of data were used as training and 30% as testing data. The scores for the accuracy, precision, recall, and F1-score metrics are very much similar to those in the previous split of data. The AUC score is the same, being the highest for the same classifier, LR, at 0.968. In the third split of data, 60% of data was used as training data and 40% as testing data. The highest score of AUC this time is slightly different, as it is 0.971 for the LR method. The last split of data shows that the highest AUC score is 0.970 for the LR method. The statistical measure, which is the kappa index, shows serious issues with data reliability. The kappa value against the highest-scoring method is still not satisfactory. It shows that data reliability, or, in other words, class balancing, regarding the minority class is needed. The AUC performance of the five classifiers is shown in Figure 5.

We can see the deviating behavior of the classifiers in Figure 5 for all splits of training data. However, it remains very much similar against each classifier in all splits of data. These AUC scores should be improved for all classifiers to obtain a robust solution to detect fraudulent activities. In the proposed framework, the solution improves the overall scores. Results achieved on the cleaned and original datasets are good but there are lower values for the AUC score. Therefore, a robust and precise solution to increase these scores is needed.

### 4.3. Experiment 2: Classification Results after Applying Outlier Normalization, Feature Selection, and Oversampling

In this experiment, the cleaned original data are provided to the outlier removal method of IQR. It removes the out-of-range values against each feature and normalizes the data. This normalized feature set is then passed to the Shapiro method, which correlates each feature to obtain a hypothesis for the normalized feature set. The highest score was 20 features used to detect fraudulent activities. Lastly, the selected features are fed to the SMOTEN method for minority and majority class equivalence. In this way, the three-step processing of data with outliers is normalized, and the selected features and oversampled feature set are further given to the five classifiers using four different splits of data, with the results described in Table 4.

In Table 3 and Table 4, the same classifiers’ performance is shown as discussed in Experiment 1. The 80–20 split of data showed the highest accuracy of 99.97 against the KNN classifier. However, the AUC score remains the highest at 1.00, which shows both classes’ equivalent behavior in classification, whereas DT shows less accuracy and a 0.99 AUC score. The kappa value is increased to more than 99%, except for GNB, which shows the statistical measure based on the goodness of the applied approach. In the second split of data, the scores are again the maximum, whereas the AUC scores are 1.00 for all classifiers. If we look at the behavior with all four splits of data, the LR’s performance in all splits is shown as 99.96 accurate, whereas it changes in the 50–50 split of data to 99.97, and this behavior is also repeated for the precision, recall, and F1-score.

**Figure 6:**
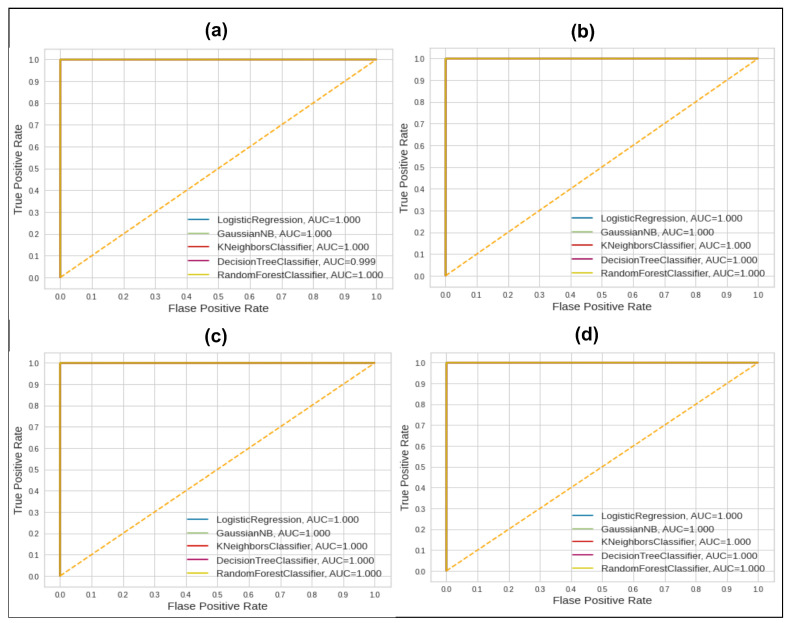
AUC scores of 5 ML classifiers after applying proposed framework techniques on 4 splits of data. (**a**) 80–20; (**b**) 70–30; (**c**) 60–40; (**d**) 50–50.

The GNB method of classification showed 93.96 accuracy and other changes in scores. GNB in the second split of data shows 93.98, whereas other measures show changes. In the third and fourth splits of data, the GNB score remains at 94.06%. The KNN classifier showed a score of 99.97 regardless of the ratio of training and testing data; the achieved scores remain the same. Similarly, DT has shown similar behavior, with 99.95% to 99.96%. Lastly, RF has shown a 99.98 score in all splits of data. The kappa value for all splits remains up to 99%, which shows the validity of the applied approach in terms of class balancing and classification results. The better performance of all classifiers remains consistent after solving the outlier issue, the feature selection using the Shapiro method, and the oversampled data using the SMOTEN method. The AUC score’s consistency is shown in Figure 6.

We can see in Figure 6 that all classifiers show the maximum AUC score of 1.00. However, we can see in Figure 5 that this behavior of the AUC values changed and it had lower values in all splits of data. This behavior of all classifiers shows that the applied techniques allow for a normalized, class-balanced, important-feature-based, precise solution. Moreover, the proposed techniques’ results are compared with those of previous studies in Table 5.

We can see that previously performed studies have given higher and much-improved results. As we can see in the first comparison, SMOTE is used for oversampling with used ML classifiers, where 97.04 AUC, accuracy, and recall are achieved, with a precision of 99.99%. In the second comparison, KNN-Coarse achieved 99.87% accuracy using text-to-image transformation, whereas ML and DL solutions of classification were used. In the third comparison, SMOTE is used for oversampling, and genetic-algorithm-based feature selection is adopted to feed ML classifiers.

This study achieved 99.82% accuracy and F1-score; furthermore, the precision reached 99.92. After looking at all the studies’ results, we can state that the proposed method is more improved than all of the other studies in terms of every measure achieved. However, the F1-score and AUC are more important measures regarding the performance of classes, which are higher in the proposed method.

## 5. Conclusions

Due to the increasing use of the Internet for buying and purchasing, credit card use has also increased. This increased usage has increased the risk of CCF. Therefore, online banking faces many cyber-attack problems and it is necessary to install a precise CCF on the transaction platform. Many studies have been conducted previously but most of them have not targeted the outlier removal and class imbalance problems before proposing ML solutions for CCF detection. However, the proposed study has given three solutions to develop a precise CCF detector. It solves the outlier problem from the Kaggle dataset, and then uses the 20 most important features using Shapiro ranking. The refined data are then used to resample the original data to solve the minority class problem. The resampled data are used to feed five ML classifiers, which leads to solving the class classification problem in a more precise way. There are two experiments conducted in which the results are collected before applying the proposed study techniques and after applying the proposed study techniques are presented. In Experiment 1, the original data were used after cleaning and fed to ML classifiers for the classification of fraudulent and non-fraudulent activities. In Experiment 2, the normalization of the outliers using the IQR method, feature selection using the Shapiro method, and oversampling using the SMOTEN method were applied. The refined and normalized data were then fed to five ML classifiers. In both experiments, holdout validation with four different splits were applied, namely 80–20, 70–30, 60–40, and 50–50. We found that in both of these experiments, the AUC score, which is more suitable for class balancing, could be used and it increased to 1.00 as compared to the Experiment 1 scores. Furthermore, the Experiment 2 results were compared with those of state-of-the-art studies, and ours was proven to be more robust, and improved method.

For future work, it is suggested to use outlier removal first, which could be applied with other methods. Furthermore, deep learning methods could be applied to different datasets of credit card fraud detection.

## Figures and Tables

**Table 1:** Summary of previously proposed credit card fraud detection studies and their achieved results.

Study	Year	Applied Methods	Dataset	Results
[25]	2019	SMOTE and other oversampling methods with classical ML models for classification	Kaggle Credit Card Fraud	Logistic regression accuracy, recall, AUC = 97.04 Precision = 99.99%
[27]	2020	Undersampling and oversampling using SMOTE, CNN and Auto-Encoder method for classification	Kaggle, private dataset	Accuracy = 93%
[28]	2021	Text to image conversion applied for data transformation; class weights are assigned to deep feature set whereas DL and ML classifiers are used for classification	Kaggle	KNN-Coarse Accuracy = 99.87%
[26]	2022	SMOTE method of oversampling with GA-FS and ML methods of classification	Kaggle	Highest achieved results using RF:accuracy = 99.82%, precision = 99.92%, F1-score = 99.82%.
[29]	2022	Credit card fraud detection using PCA transformation and random under- and oversampling methods with SMOTE and classification using random forest	Data Card Fraud of Europe	Testing accuracy = 99.997%

**Table 2:** Data distributions of original and oversampled datasets.

Methods	Normal	Fraudulent	Total
Original	284,315	492	284,807
Oversampled	284,315	284,315	568,630

**Table 3:** Classification results on 4 different splits of original cleaned data.

Split	Method	Accuracy (%)	Recall (%)	Precision (%)	F1-Score (%)	AUC	Kappa
80–20	LR	99.91	99.91	99.90	99.90	0.968	76.10
	GNB	97.91	97.91	99.80	98.79	0.964	10.84
	KNN	99.95	99.95	99.95	99.95	0.923	86.79
	DT	99.92	99.92	99.91	99.91	0.862	77.91
	RF	99.95	99.95	99.95	99.95	0.948	86.65
70–30	LR	99.90	99.90	99.89	99.90	0.968	73.69
	GNB	97.75	97.75	99.79	98.70	0.946	10.71
	KNN	99.95	99.95	99.94	99.94	0.912	82.15
	DT	99.91	99.91	99.91	99.91	0.876	73.11
	RF	99.94	99.94	99.94	99.94	0.939	81.29
60–40	LR	99.92	99.92	99.91	99.91	0.971	73.26
	GNB	97.79	97.79	99.81	98.73	0.964	11.99
	KNN	99.94	99.94	99.94	99.94	0.924	82.69
	DT	99.93	99.93	99.93	99.93	0.881	76.84
	RF	99.95	99.95	99.95	99.95	0.941	84.05
50–50	LR	99.91	99.91	99.90	99.90	0.970	72.21
	GNB	97.86	97.86	99.80	98.76	0.956	11.02
	KNN	99.94	99.94	99.94	99.94	0.915	82.27
	DT	99.89	99.89	99.90	99.90	0.866	75.27
	RF	99.94	99.94	99.94	99.94	0.944	85.44

**Table 4:** Classification results on 4 different splits of normalized outliers, selected features, and oversampled data.

Split	Method	Accuracy (%)	Recall (%)	Precision (%)	F1-Score (%)	AUC	Kappa
80–20	LR	99.96	99.96	99.96	99.96	1.00	99.91
	GNB	93.97	93.97	94.62	93.95	1.00	88.13
	KNN	99.97	99.97	99.97	99.97	1.00	99.94
	DT	99.95	99.95	99.95	99.95	0.99	99.90
	RF	99.98	99.98	99.98	99.98	1.00	99.95
70–30	LR	99.96	99.96	99.96	99.96	1.00	99.92
	GNB	93.98	93.98	94.62	93.96	1.00	88.10
	KNN	99.97	99.97	99.97	99.97	1.00	99.94
	DT	99.95	99.95	99.95	99.95	1.00	99.92
	RF	99.98	99.98	99.98	99.98	1.00	99.95
60–40	LR	99.96	99.96	99.96	99.96	1.00	99.92
	GNB	94.06	94.06	94.68	94.04	1.00	87.95
	KNN	99.97	99.97	99.97	99.97	1.00	99.94
	DT	99.96	99.96	99.96	99.96	1.00	99.91
	RF	99.98	99.98	99.98	99.98	1.00	99.96
50–50	LR	99.97	99.97	99.97	99.97	1.00	99.93
	GNB	94.06	94.06	94.68	94.04	1.00	88.02
	KNN	99.97	99.97	99.97	99.97	1.00	99.94
	DT	99.95	99.95	99.95	99.95	1.00	99.90
	RF	99.98	99.98	99.98	99.98	1.00	99.95

**Table 5:** Comparison of proposed work with state-of-the-art studies.

Study	Year	Methods	Dataset	Results
[25]	2019	SMOTE and other oversampling methods with classical ML models for classification	Kaggle	Logistic regression accuracy, recall, AUC = 97.04 Precision = 99.99
[28]	2021	Text to image conversion applied for data transformation; class weights are assigned to deep feature set, whereas DL and ML classifiers are used for classification	Kaggle	KNN-Coarse Accuracy = 99.87%
[26]	2022	SMOTE method of oversampling with GA-FS and ML methods of classification	Kaggle	Highest achieved results using RF:accuracy = 99.82%, precision = 99.92%, F1-score = 99.82%.
Proposed		Outliers normalized using IQR, feature ranking using Shapiro, oversampling using SMOTEN, and 5 ML classifiers used for classification	Kaggle	Highest accuracy = 99.98, precision = 99.98, recall = 99.98, AUC = 1.00, kappa = 99.95

## Data Availability

The dataset is publicly available.
